# Maternal undernutrition alters the skeletal muscle development and methylation of myogenic factors in goat offspring

**DOI:** 10.5713/ab.21.0285

**Published:** 2022-01-03

**Authors:** Xiaoling Zhou, Qiongxian Yan, Liling Liu, Genyuan Chen, Shaoxun Tang, Zhixiong He, Zhiliang Tan

**Affiliations:** 1 College of Animal Science, Tarim University, Alaer 843300, China; 2 Institute of Subtropical Agriculture, The Chinese Academy of Sciences, Changsha 410125, China; 3 College of Resources and Environment, University of Chinese Academy of Science, Beijing 100049, China; 4 Hunan Co-Innovation Center for Utilization of Botanical Functional Ingredients, Changsha 410128, China; 5 Hunan Co-Innovation Center of Animal Production Safety, CICAPS, Changsha 410128, China

**Keywords:** DNA Methylation, Goat, Intrauterine Undernutrition, Muscle Fiber Type, Myogenic Factors

## Abstract

**Objective:**

The effects of maternal undernutrition during midgestation on muscle fiber histology, myosin heavy chain (MyHC) expression, methylation modification of myogenic factors, and the mammalian target of rapamycin (mTOR) signaling pathway in the skeletal muscles of prenatal and postnatal goats were examined.

**Methods:**

Twenty-four pregnant goats were assigned to a control (100% of the nutrients requirement, n = 12) or a restricted group (60% of the nutrients requirement, n = 12) between 45 and 100 days of gestation. Descendants were harvested at day 100 of gestation and at day 90 after birth to collect the femoris muscle tissue.

**Results:**

Maternal undernutrition increased (p<0.05) the fiber area of the vastus muscle in the fetuses and enhanced (p<0.01) the proportions of MyHCI and MyHCIIA fibers in offspring, while the proportion of MyHCIIX fibers was decreased (p<0.01). DNA methylation at the +530 cytosine-guanine dinucleotide (CpG) site of the myogenic factor 5 (*MYF5*) promoter in restricted fetuses was increased (p<0.05), but the methylation of the *MYF5* gene at the +274,280 CpG site and of the myogenic differentiation (*MYOD*) gene at the +252 CpG site in restricted kids was reduced (p<0.05). mTOR protein signals were downregulated (p<0.05) in the restricted offspring.

**Conclusion:**

Maternal undernutrition altered the muscle fiber type in offspring, but its relationship with methylation in the promoter regions of myogenic genes needs to be elucidated.

## INTRODUCTION

During myogenesis, the numbers of myoblasts, primary muscle fibers, and secondary muscle fibers are determined before birth in humans [1] and domestic animals [2]. Skeletal muscle is highly susceptible to nutritional deficiencies due to the low priority of nutrient acquisition in mammals [3,4]. Nutritional deficiency in early gestation decreases the number of primary muscle fibers [5], but the effects are generally minor due to the low nutrients requirement of early fetuses. Secondary muscle fiber proliferates from approximately 0.3 G (30% of gestation stage), and this process ceases at approximately 0.7 G [1,2,6], which constitutes nearly 95% of the final fiber population [6]. Insufficient nutrition during midgestation (0.3 to 0.7 G) affects the number, size, and type of secondary fibers before [6,7] and after birth [8,9]. It is widely accepted that skeletal muscle development is nutritionally programmed *in utero* [3,4], and knowledge of the underlying mechanisms of this programming helps to understand the nosogenesis of some congenital myopathies, such as fiber type disproportion in humans, or to improve meat production in livestock.

Alterations in muscle phenotype can partly be interpreted by changes in hormone response [10], the mammalian target of rapamycin (mTOR) signaling pathway [7], myogenic regulatory factor expression [11,12] and noncoding RNA [13]. However, an unresolved question is how the phenotype is programmed during ontogenetic muscle development. Epigenetic modification is one of the key mechanisms responsible for persistent programming [3]. Myogenic factors, including myogenic differentiation 1 (MYOD), myogenin (MYOG), myogenic factor 5 (MYF5), and myogenic factor 6 (MYF6), govern skeletal muscle determination and differentiation [14], and among these, MYF5 and MYOD are the master regulators. Epigenetic modification, including DNA methylation of enhancer chromatin [15], histone acetylation, and long noncoding RNA [13] of the *MYF5* and *MYOD* genes, is associated with the expression of muscle-specific genes during myogenesis and differentiation. Methylation of the promoter region is a regulatoty target in intrauterine skeletal programming, for example methylation of the peroxisome proliferator-activated receptor gamma coactivator-1 alpha (*PGC1A*) promoter [16]. However, the relationship between methylation of the promoter regions of the *MYF5* and *MYOD* genes and uscle developmental programming under nutrient stress remains uncharacterized. DNA methylation generally reduces the expression of muscle-related genes [16,17]. We thus hypothesized that maternal undernutrition during midgestation would decrease the expression of *MYF5* and *MYOD* genes by increasing the methylation of their promoter regions.

Small ruminants are optimum animal models for studying intrauterine programming, and 40% energy restriction in pregnant goats led to hepatic metabolic programming in offspring in our previous study [18]. In the present study, we investigated the effects of 40% maternal undernutrition during midgestation on muscle fiber histology, myosin heavy chain expression, methylation modification of myogenic factors, and the mTOR signaling pathway in the skeletal muscles of prenatal and postnatal goats.

## MATERIALS AND METHODS

### Animal care

The present experiment was reviewed and approved by the Animal Care Committee according to the Animal Care and Use Guidelines of the Institute of Subtropical Agriculture, the Chinese Academy of Sciences, Changsha, China (No. KYNEAAM-2015–0009).

### Experimental design and animal management

Twenty-four goats (45±3 days of gestation, Liuyang black goats, a local breed) were selected and tested by portable ultrasonography (Aloka SSD-500 with a 5-MHz linear probe; Aloka, Shanghai, China). Dams were randomly assigned to the control (100% of the nutrients requirements suggested in the Chinese Meat Goat Requirements [2004], n = 12) or restricted group (60% of the nutrients requirements, n = 12) according to body weight (BW) and litter size. All dams were placed within individual pens and fed a diet twice (0800 h and 1600 h) per day with a 50:50 ratio of concentrate to roughage, with free access to drinking water. The ingredients and composition of the experimental diet on a dry matter basis are shown in additional Supplementary Table S1. The feeding of the restricted group was conducted by providing 60% of the feed allowance of the control group from 45 to 100 days of gestation, and the actual restriction level (1.04 kg/d for the control group vs 0.62 kg/d for the restricted group) was 60.2% after measurement of the daily feed allowance and refusal. At day 100 of gestation, six pregnant dams per group were selected for harvesting, and 10 fetuses (three singlets, two sets of twins, and one set of triplets) in each group were obtained. The ratio of females to males was 7:3 in the control group and 6:4 in the restricted group.

After day 100 of gestation, feed restriction was removed, and the remaining dams were fed to supply 100% of the nutrients requirements and were managed as before during the following gestation and lactation stages. The length of the gestation period was approximately 150 days. After parturition, neonatal kids were nursed by their dams until preweaning between days 50 and 60. Preweaning was conducted by separating offspring from their dams in the daytime from 0800 to 1600 h, and a mixed diet of starter and fresh *Miscanthus* spp. was provided at a ratio of 20:80 during this period. Complete weaning was performed at 60 days of age. Then, all kids were given *ad libitum* access to the above diet and had free access to drinking water. The ingredients and composition of the kid diet are shown in additional Supplementary Table S1. At 90 days of age, eight kids in each group were obtained for harvesting. The ratio of females to males was 3:5 in the control group and 4:4 in the restricted group.

#### Body weight measurement and muscle tissue sampling

At day 100 of gestation and at day 90 after birth, the feeding of all selected animals was withdrawn for 24 h, while clear water was offered freely. Following electric shock and exsanguination, fetuses at day 100 were removed from the uterus. After the umbilical cord was cut, each fetus was immediately weighed. The empty BW of kids was reported previously [18]. Samples of semitendinosus (ST) and vastus lateralis (VL) muscle were collected from the kids, while the gross vastus muscle in the fetuses was sampled because it was difficult to dissect into individual muscles. The sliced tissue samples were immersed in 10% formalin fixing solution for histology measurement, while another aliquot of samples was quick-frozen in liquid nitrogen and then stored at −80°C for further analysis.

### Histology

After fixation, the muscle samples were embedded in paraffin, sliced at a thickness of 8 μm using a rotary microtome (RM2016; Leica Microsystems Inc., Wetzlar, Hesse-Darmstadt, Germany), and stained with hematoxylin (AS1055A; Aspen Biological, Inc., Wuhan, China) and eosin (AS1094; Aspen Biological, Inc., China) according to Zou et al [11]. Ten different microscopic fields of each section and five sections per sample were randomly chosen to determine the muscle fiber area using the Image-Pro Plus 6.0 software (Media Cybernetics, Bethesda, MD, USA).

### Quantitative real-time polymerase chain reaction

The extraction and quantification of total RNA and the analyses of messenger RNA (mRNA) expression levels were performed using the SYBR green-based method with gene-specific primers (Table 1) according to Zhou et al [18]. A melting curve analysis was conducted to confirm specific product amplification. Actin gamma 1 (*ACTG1*) was used as the reference gene, and the real-time polymerase chain reaction data were calculated using the 2^−ΔΔCt^ method. The expression of mRNA is presented as the fold change relative to the reference gene.

### Immunoblotting analysis

The extraction and quantification of total protein and the immunoblotting analysis were carried out according to the method described previously [19]. The primary antibodies against MyHCI (MYH7, No. MFCD00162703; Merck KGaA, Darmstadt, Hesse-Darmstadt, Germany), MyHC-IIa (MYH2, No. ab124937; Abcam PLC, Cambridge, MA, USA), MyHC-IIx (No. BM0096; Boster Biological Technology Co., Ltd, Pleasanton, CA, USA), and β-tubulin (No. 2146S; Cell Signaling Technology, Inc., Danvers, MA, USA) were diluted 1:5,000, 1:10,000, 1:100, and 1:1,000, respectively. Primary antibodies against AKT serine/threonine kinase 1 (AKT, No. AV06008; Merck KGaA, Germany), p-AKT (No. 9275; Cell Signaling Technology, Inc., USA), phosphoinositide-3-kinase regulatory subunit 1 (PI3K, No. C312573; LifeSpan BioSciences, Inc., Seattle, WA, USA), p-PI3K (No. C358831; LifeSpan BioSciences, Inc., USA), mTOR (No. PLA0114; Merck KGaA, Germany), p-mTOR (No. 2971; Cell Signaling Technology, Inc., USA), and β-actin (No. 4976; Cell Signaling Technology, Inc., USA) were diluted as 1/1,000. The density of bands was quantified and then normalized to the reference protein of β-tubulin or β-actin. The normalized values were used for comparison of the relative expression levels of the target proteins between the control group and the restricted group.

### DNA methylation detection

Approximately 20 mg of frozen sample was ground in liquid nitrogen, and DNA was extracted using a DNeasy Blood & Tissue Kit (Qiagen, Hilden, Nordrhein-Westfalen, Germany) according to the manufacturer’s instructions. Quantitative methylation analysis of multiple cytosine-guanine dinucleotide (CpG) sites was performed by the Beijing Genomics Institute (Guanzhou, China) using a Sequenom EpiTYPE system based on MALDI-TOF mass spectrometry according to Suchiman et al [20]. The CpG-rich sequences for *MYF5* and *MYOD* promoters were selected using EMBOSS Cpgplot (https://www.ebi.ac.uk/Tools/seqstats/emboss_cpgplot/), and one CpG island was found in the promoter regions of *MYF5* and *MYOD*. The CpG island of the *MYF5* promoter ranges from −10 to +347 bp relative to the transcriptional start site (TSS) (10070240–10070597 on chromosome 5). The forward primer sequence of *MYF5* was TTTATTTTGGG TAGTTTTTGGTTAGG tagged with the T7 promoter sequence aggaagagag, while the reverse sequence was CCCA AAAATATATAAAAAACCCCAA tagged with the sequence cagtaatacgactcactatagggagaaggct. The product size was 558 bp from −44 to +503 (10070196 to 10070753 on chromosome 5) and covered 41 CpG sites, among which 34 CpGs could be effectively quantified. The CpG island of the *MYOD* promoter ranges from +159 to +888 relative to the TSS site (47532862 to 47533591 on chromosome 15). The forward primer sequence of *MYOD* was TAGTTTTGGGAGTTTA GTGTGAAGG tagged with the T7 promoter sequence agga agagag, while the reverse sequence was CCTTACAAACCC ACAATAAACAA was tagged with cagtaatacgactcactatag ggagaaggct. The product size was 546 bp from −15 to +530 (47532688–47533233 on chromosome 15) and covered 55 CpG sites, among which 42 CpG sites could be effectively quantified. The spectral data were preprocessed and analyzed according to the method of Suchiman et al [20]. The methylation level of the restricted group in each CpG site was expressed as the value relative to the control group.

### Statistical analysis

Data were analyzed by a mixed model with treatment, gender, and litter size as fixed factors, and initial BW of dams as the covariate. Statistical significance was considered at p<0.05, and the Sidak method was applied to compare mean values. All results are presented as the mean and standard errors.

## RESULTS

### Body weight and area of muscle fibers

**Figure 1:**
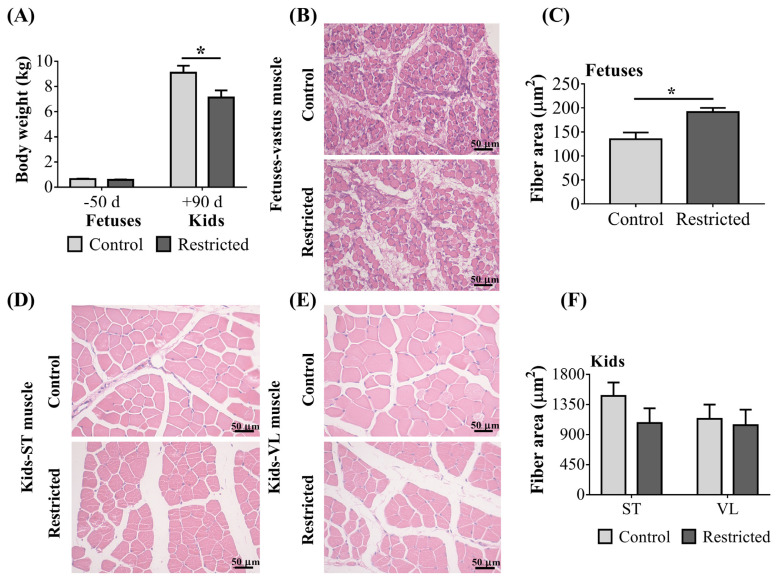
Body weight and muscle fiber area in offspring. (A) Body weight; (B) histological section or (C) fiber area of vastus muscle in fetuses; (D) histological section of semitendinosus (ST) muscle or (E) vastus lateralis (VL) muscle in kids; (F) fiber area of ST and VL muscles in kids. * p<0.05.

The effect of maternal undernutrition on the muscle fiber area of fetuses and kids is presented in Figure 1. The BW of fetuses was unaffected (p>0.05) by undernutrition, but the BW of restricted kids was decreased relative to controls (p = 0.027).

The muscle fiber area of the vastus muscle of restricted fetuses was increased (p = 0.032), while it was unaffected (p> 0.05) in the ST and VL muscles of restricted kids. The Gender and litter size did not affect the area of muscle fibers in the fetuses or kids (p>0.05).

### Myosin heavy chain expression

**Figure 2:**
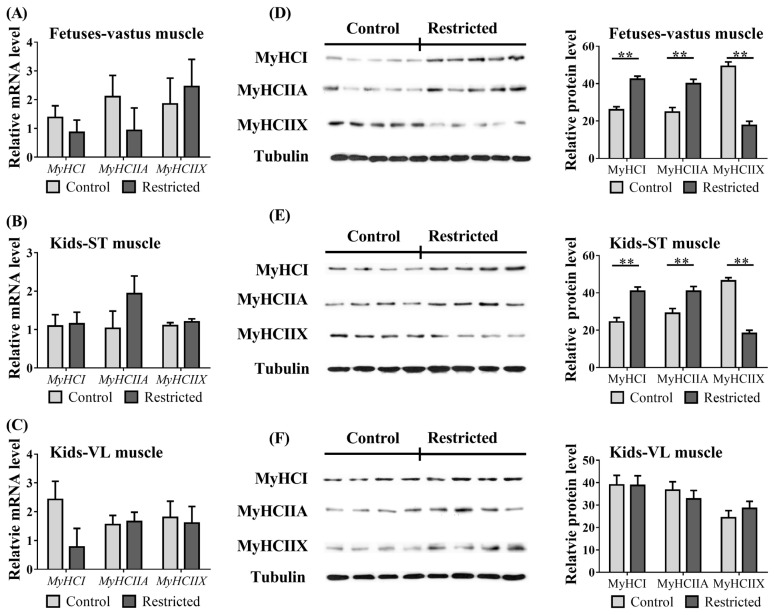
The mRNA and protein expression of myosin heavy chain in offspring. (A) mRNA expression of vastus muscle in fetuses; (B) mRNA expression of semitendinosus (ST) or (C) vastus lateralis (VL) muscle in kids; (D) protein expression of vastus muscle in fetuses; (E) protein expression of ST or (F) VL muscle in kids. ** p<0.01.

The effect of maternal undernutrition on the expression of myosin heavy chain (MyHC) in the muscle tissues of fetuses and kids is presented in Figure 2. The expression of *MyHCIIB* mRNA in the fetuses and kids was not detectable, so the expression of MyHCIIB protein in these samples was not analyzed further. Compared to the control group, maternal undernutrition did not change (p>0.05) the expression of *MyHCI*, *MyHCIIA*, or *MyHCIIX* mRNA in the vastus muscles of fetuses (Figure 2A) or in the ST (Figure 2B) and VL (Figure 2C) muscles of kids. However, the protein expression levels of MyHCI and MyHCIIA were increased (p<0.05) both in the vastus muscles of fetuses (Figure 2D) and in the ST muscles of kids (Figure 2E), while the MyHCIIX protein expression in the fetuses and in the ST muscles of kids was decreased (p<0.01). Protein expression levels of MyHCI, MyHCIIA, and MyHCIIX in the VL muscles of kids (Figure 2F) were not affected (p>0.05). Gender and litter size did not affect (p>0.05) the mRNA or protein expression of *MyHCI*, *MyHCIIA*, and *MyHCIIX* genes in the fetuses or kids.

### mRNA expression and DNA methylation of myogenic factors

**Figure 3:**
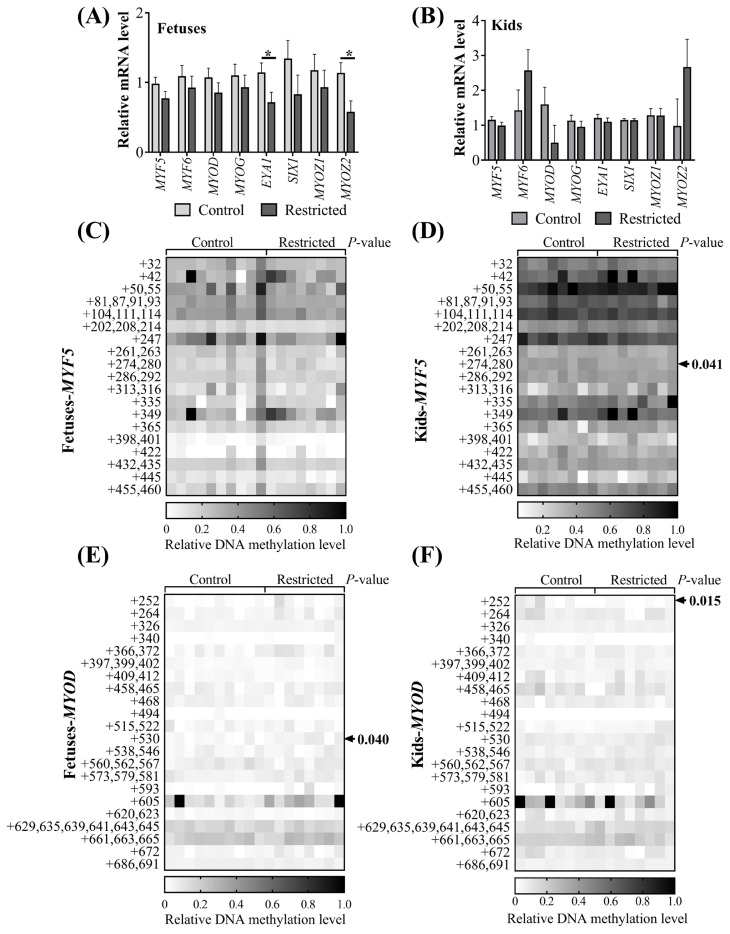
The mRNA expression of myogenic genes and CpG methylation of myogenic promoters in offspring. (A) mRNA expression of vastus muscle in fetuses; (B) mRNA expression of semitendinosus (ST) muscle in kids; methylation of CpG sites in the (C) *MYF5* or (E) *MYOD* promoter region relative to the TSS site in vastus muscle of fetuses; methylation of CpG sites in the (D) *MYF5* or (F) *MYOD* promoter region relative to the TSS site in the semitendinosus (ST) muscle of kids. *MYF5*, myogenic factor 5; *MYOD*, myogenic differentiation. * p<0.05.

Since significant differences in myosin heavy chain protein expression in muscle resulting from maternal undernutrition were observed, we further determined the mRNA expression and DNA methylation of myogenic factors in the muscles of fetuses and kids (Figure 3). Maternal undernutrition did not influence (p>0.05) the expression of *MYF5*, *MYF6*, *MYOD*, or *MYOG* mRNA in the vastus muscles of fetuses (Figure 3A) or in the ST muscles of kids (Figure 3B). The expression levels of eyes absent homolog 1 (*EYA1*) (p = 0.044) and myozenin 2 (*MYOZ2*) (p = 0.038) mRNA in the vastus muscles of restricted fetuses were downregulated, while no difference was observed in the ST muscles of kids (p>0.05). The methylation level of the CpG island of the *MYF5* promoter was not affected (p>0.05) in the fetuses (Figure 3C). Maternal undernutrition increased (p = 0.040) the methylation level of the +530 CpG site of the *MYOD* promoter in the fetuses (Figure 3D), while the methylation levels of the +274, 280 CpG site of the *MYF5* promoter (Figure 3E) and the +252 CpG site of the *MYOD* promoter (Figure 3F) in the restricted kids were decreased (p<0.05). Gender and litter size did not affect (p> 0.05) the mRNA expression of these myogenic genes or the CpG methylation of *MYF5* and *MYOD* promoters in the fetuses. Gender affected (p<0.05) the *MYF5* and SIX homeobox 1 (*SIX1*) mRNA expression in kids; both was greater in females than in males. The methylation of the +32 CpG site in the *MYF5* promoter of the female kids was higher (p = 0.041) than in the males.

### mTOR signaling pathway

**Figure 4:**
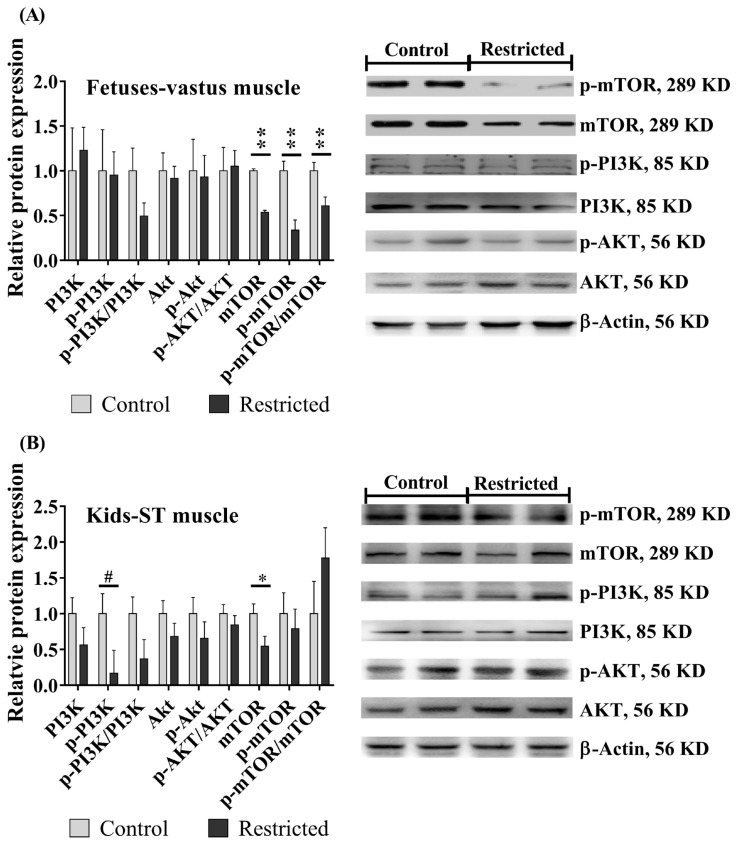
The protein expression of mammalian target of rapamycin (mTOR) signaling in the skeletal muscles of offspring. (A) Vastus muscles in fetuses; (B) semitendinosus (ST) muscles in kids. ^#^ 0.05<p<0.10; * p<0.05; ** p<0.01.

Maternal undernutrition reduced (p<0.01) the protein expression of mTOR, p-mTOR, and the ratio of p-mTOR/mTOR in fetal vastus muscle (Figure 4A). Maternal undernutrition also reduced (p = 0.037) the mTOR protein expression in kids and tended to reduce (p = 0.088) the PI3K phosphorylation level (Figure 4B), while the effects on the PI3K, AKT, and p-AKT proteins and the ratio of p-AKT/AKT in kids were not significant (p>0.05). Gender and litter size did not affect the above protein expression in the fetuses or kids (p> 0.05).

## DISCUSSION

During the past two decades, there has been increased research interest in the effects of maternal undernutrition on muscle development in offspring. In this study, maternal undernutrition altered the muscle fibers and MyHC types of the vastus muscle in fetuses and kids, and downregulated the mRNA expression of myogenic factors in the fetuses. DNA methylation levels of several sites in the *MYF5* and *MYOD* promoter regions in the fetuses and kids were also affected by maternal undernutrition. Several studies have confirmed that maternal undernutrition in the second trimester is closely associated with the reduced weight of offspring in human epidemiological surveys [21] and in animal model trials [8,22]. In the present study, no changes in the BW of fetuses and the diminished BW in restricted kids were consistent with a compensation effect by the mother *in utero* and a thrifty phenotype after birth. However, the increase in muscle fiber area in restricted fetuses in this study was not consistent with the findings (no significant change) in the fetuses of cattle [12] and pigs [11] during midgestation or in sheep (a decreased size) [7]. Whether this phenomenon is due to species differences or other physiological factors needs more in-depth research. Notably, the perimysium area of the ST and VL muscles seemed larger in the restricted kids. This fraction contains a mixture of mesenchymal stem or stromal cells, fibroblasts, immune cells and endothelial cells [23]. Regrettably, we did not carry out in-situ sampling. The process of cutting muscle after slaughter may cause displacement between muscle bundles, so it is difficult to judge whether the thicker perimysium is caused by the experimental treatment or muscle sampling, hence no statistical analysis of this data has been carried out.

Skeletal muscle is generally divided into four types according to MyHC isoforms: slow-twitch type I (MyHCI) and fast-twitch types IIA (MyHCIIA), IIB (MyHCIIB), and IIX (MyHCIIX). Previously, Zhu et al [9] reported that 50% maternal undernutrition increased the MyHCIIB ratio and decreased the percentage of MyHCIIA of the longissimus dorsi (LD) muscle in restricted lambs at eight months of age, while the MyHC type of LD and ST muscles in 50% restricted lambs at 150 days of age was unaffected [8]. In contrast, Fahey et al [24] reported fewer fast fibers and more slow fibers in the LD and VL muscles of 50% maternally restricted 14-day-old lambs. In this study, the types of MyHCI and MyHCIIA were increased, but MyHCIIX was decreased both in the vastus muscle of 40% restricted fetuses and in the ST muscles of restricted kids, while the VL muscles in offspring were not affected. These results suggest that the effects of similar maternal undernutrition during midgestation on muscle fiber development may vary, and the cause needs further research. Moreover, comparing LD and ST fibers from the same muscle position, a similar degree of feeding restriction (such as 50%) exerted different effects on muscle fiber development [8,9,24]. It has been speculated that this discrepancy is related not only to the MyHC types in different muscle positions but also to the timing of phenotypic plasticity induced by different types of nutrient restriction [25].

In this study, we noted an inconsistency in the expression between the mRNA and protein levels of MyHC isoforms in the fetal vastus and kid ST muscles. Similar differences in mRNA and protein abundances of *MyHC* genes have been reported in other species [26]. Generally, the protein accretion rate of hindlimb muscle is approximately 0.17% per day in ovine fetuses [27] and 0.8% in young lambs [28]. However, transcriptional and posttranscriptional regulation of *MyHC* isoform mRNA in muscle tissue is active and sensitive to environmental cues (e.g., hormones or metabolites) [5,10]. MyHC expression at the protein level represents the existing fiber type within the muscle. Therefore, the effect of protein expression is highlighted.

We observed changes in the MyHC types of vastus muscle in goat offspring. The results hinted that metabolic programming could have occurred in the present study. Hence, we determined the effect on the DNA methylation of promoter sites in the *MYF5* and *MYOD* genes. DNA methylation is one of the universal mechanisms regulating this type of phenotypic change [17]. *MYF5* and *MYOD* gene methylation is important in regulating muscle differentiation. Increased methylation of three important CpG sites in the *MYOD* gene and decreased *MYOD* mRNA expression have been observed during the differentiation of human primary muscle fibers from myoblasts to myotubes, while the methylation and mRNA levels of the *MYF5* gene were decreased [29]. However, there are few reports on the methylation modification of these two myogenic regulatory factors under intrauterine nutrition restriction. Our results revealed that maternal nutritional restriction during midgestation affected the methylation of individual sites in the *MYOD* and *MYF5* promoter regions of the fetuses and kids. These affected sites may not be closely correlated with the regulation of *MYOD* and *MYF5* mRNA expression, because the mRNA expression levels of *MYOD* and *MYF5* were unaffected. Previous studies have shown that maternal low-amino acid diets in the middle and late stages of pregnancy reduced the methylation level of the CpG island in insulin like growth factor 2 receptor (*IGF2R*) gene intron 2 in the longissimus muscles of sheep fetuses at 130 days [30]. A similar study found that the average CpG island methylation in the *PGC1A* promoter sequence in the skeletal muscle of intrauterine growth retardation rats with protein restriction during pregnancy increased, while glucose transporter type 4 and *PGC1A* mRNA expression and glucose tolerance decreased [16]. In this study, we have not yet identified the key methylation modification sites that control the phenotypic changes in offspring; thus, further research is needed.

The mRNA expression levels of the myogenic factors *MYOG* and *MRF4* in fetuses and kids were unaffected, but the mRNA levels of *EYA1* and *MYOZ2* were decreased in the restricted fetuses. The *SIX1* and *EYA1* genes are specifically involved in the differentiation of limb buds [31]. The SIX1 and EYA1 proteins accumulate preferentially in the nuclei of fast-twitch muscles and induce a fiber-type transition with the replacement of myosin heavy chain I and IIA isoforms by the faster IIB and/or IIX isoforms [32]. Furthermore, MYOZ2 is expressed in slow-twitch skeletal muscle, and the downregulation of MYOZ2 is associated with increased slow-twitch muscle fibers [33]. The downregulation of *EYA1* and *MYOZ2* mRNA in restricted fetuses is consistent with the increases in MyHCI and MyHCIIA types and the decrease in MyHCIIx in the present study.

The development of skeletal muscle is a highly complicated process involving nutritional factors and numerous signaling pathways that regulate muscle-specific transcription factors, and mTOR signaling is a key regulator of skeletal muscle development at distinct stages of myogenesis [34]. In this study, the mTOR protein was downregulated in the femoris muscles of fetuses and kids. We considered that compared to the normal energy requirement, 40% maternal undernutrition aggravated the overall lack of proteins and amino acids, resulting in the downregulation of mTOR signals in the offspring, thereby affecting the protein synthesis of muscle tissues. Furthermore, the mTOR protein controls muscle-specific miR-1 transcription in mice, which regulates the stability of *MYOD* and *MYF5* expression [35]. In addition, other miRNAs such as miR-133, miR-206, and miR125b are also linked to the mTOR pathway to regulate muscle fiber differentiation [35], and the muscle-specific expression of miRNAs can be regulated by nutrients through epigenetic mechanisms [34]. This provides new ideas for studying the mechanism of muscle fiber development programming under maternal nutrition restriction.

## CONCLUSION

Maternal undernutrition during midgestation increased the expression of MyHCI and MyHCIIA proteins in the vastus muscles of fetuses and the ST muscles of kids and reduced MyHCIIX protein expression, hinting at the developmental programming of skeletal muscle fibers. Transcripts of *EYA1* and *MYOZ2* genes in restricted fetuses were downregulated, and mTOR protein signals declined in restricted fetuses and kids. Maternal feeding restriction may prompt the switch from MyHCIIX to MyHCI and IIA in the femoris muscles of fetuses and kids associated with repression of the mTOR pathway. This programming was associated with the reduction of EYA1 and MYOZ2 transcripts, but the association between mRNA expression and DNA methylation in the promoter region of the myogenic factors *MYF5* and *MYOD* needs to be further investigated.

## Figures and Tables

**Table 1:** Primer sequences used for quantitative real-time polymerase chain reaction

Gene	Forward (5′ to 3′)	Reverse (5′ to 3′)	Product length (bp)	Locus
*MyHCI*	ACCAACCTGTCCAAGTTCCG	CGCGGCTACTCCTCATTCAA	143	XM_018054604.1
*MyHCIIA*	AAGGGCTGACATTGCTGAGT	TGCCTCTCTTCAGTCATTCCA	122	XM_018064659.1
*MyHCIIX*	GGTCTACGCAAACACGAGAG	GCGGAATTTGGAGAGGTTGAC	177	XM_018064657.1
*MYF5*	AGACGCCTGAAGAAGGTCAA	CTCCACCTGTTCCCTTAGCA	150	NM_001287037.1
*MYF6*	CAAGTCAGAGGCCAAGGAAG	TTCTAAGGGCTGCAGGGTAA	103	NM_001285602.1
*MYOD*	TGCAAACGCAAGACGACTAA	CTGGTTTGGGTTGCTAGACG	126	XM_018058990.1
*MYOG*	ACAATCTGCACTCCCTCACC	CATCCTGGCAGACAATCTCA	106	NM_001285733.1
*EYA1*	CCACTCATGTCCAGCTCAGA	GACTGCGAGGCTGTTAAACC	137	XM_013967225.2
*SIX1*	CAGTCACCTCGCACTTTGAA	TCCTTCATTTCCCACAGAGG	160	XM_018058424.1
*MYOZ1*	GGACAGCAATGCCTTATGGT	AACTAAGGGTTCGCTCAGCA	101	XM_005699215.3
*MYOZ2*	TGCCATGCAGAATGAGAAAC	TAGGGACAGCTGTGGTGTTG	182	XM_013964451.2
*ACTG1*	ATGGCTACTGCTGCGTCGT	TTGAAGGTGGTCTCGTGGAT	161	XM_018063603.1

*MyHCI*, myosin heavy chain 1; *MyHCIIA*, myosin heavy chain IIA; *MyHCIIX*, myosin heavy chain IIX; *MYF5*, myogenic factor 5; *MYF6*, myogenic factor 6; *MYOD*, myogenic differentiation 1; *MYOG*, myogenin; *EYA1*, eyes absent homolog 1; *SIX 1*, homeobox protein SIX1; *MYOZ1*, myozenin 1; *MYOZ2*, myozenin 2; *ACTG1*, actin gamma 1.

## SUPPLEMENTARY MATERIAL

**Supplementary Table S1**. Ingredients and composition of experimental diets for dams and kids
