# Supplementary material for: Maternal undernutrition alters the skeletal muscle development and methylation of myogenic factors in goat offspring
Source: Anim Biosci. 2022 Jan 3;35(6):847–57. doi: 10.5713/ab.21.0285 (PMC9066034; doi:10.5713/ab.21.0285)
Supplement: Supplementary file 1 [file ab-21-0285-suppl.pdf]

Additional Table S1 Ingredients and composition of experimental diets for dams and kids (DM basis)

Item	Dams	Kids
Ingredients (%)		
Miscanthus	50.00	20.00
Maize	33.50	36.00
Wheat bran	-	14.40
Soybean meal	10.33	14.16
Whey power	-	6.40
Fat power	4.00	6.40
Calcium carbonate	0.49	0.24
Calcium hydrophosphate	0.46	0.80
Sodium chloride	0.22	0.40
Premix[†]	1.00	1.20
Composition		
Metabolic energy (MJ/kg)	11.78	15.19
Crude protein (%)	12.05	15.52
Ether extract (%)	8.97	10.46
Neutral detergent fiber (%)	64.44	24.99
Acid detergent fiber (%)	28.32	11.67
Ash (%)	5.89	3.59
Calcium (%)	0.53	0.76

Phosphorus (%)	0.20	0.32

†Contained per kg of diet: 1.19 g MgSO4•H2O, 25 mg FeSO4•7H2O, 8 mg CuSO4•5H2O, 30 mg MnSO4•H2O, 50 mg ZnSO4•H2O, 0.1 mg Na2SeO3, 0.4 mg KI, 0.3 mg CoCl2•6H2O, 950 IU vitamin A, 175 IU vitamin D, and 180 IU vitamin E. ME was calculated according to [1]. The rest are measured values.

References

1. Zhang HF. Nutrition parameters and feeding standard for animals. 2nd ed. Beijing: China agriculture press; 2010.
